# Interferon signaling in ascites-associated macrophages is linked to a favorable clinical outcome in a subgroup of ovarian carcinoma patients

**DOI:** 10.1186/s12864-017-3630-9

**Published:** 2017-03-21

**Authors:** Till Adhikary, Annika Wortmann, Florian Finkernagel, Sonja Lieber, Andrea Nist, Thorsten Stiewe, Uwe Wagner, Sabine Müller-Brüsselbach, Silke Reinartz, Rolf Müller

**Affiliations:** 1 Institute of Molecular Biology and Tumor Research (IMT), Center for Tumor Biology and Immunology (ZTI), Philipps University, Marburg, Germany; 2 Genomics Core Facility, Center for Tumor Biology and Immunology (ZTI), Philipps University, Marburg, Germany; 3 Clinic for Gynecology, Gynecological Oncology and Gynecological Endocrinology, Center for Tumor Biology and Immunology (ZTI), Philipps University, Marburg, Germany

**Keywords:** Ovarian cancer ascites, Tumor-associated macrophages, Interferon signaling, CD163, Interleukin 6, Interleukin 10, *PCOLCE2*

## Abstract

**Background:**

Although tumor-associated macrophages (TAMs) are essential for cancer progression, connections between different clinical outcomes and transcriptional networks have not been reported. We have addressed this issue by analyzing global expression patterns of TAMs isolated from the ascites of ovarian cancer patients.

**Results:**

TAMs isolated from different ovarian cancer patients can be stratified by coexpression or principal component analysis into subgroups with specific biological features and associated with distinct clinical outcomes. A hallmark of subgroup A is a high expression of clinically unfavorable markers, including (i) high CD163 expression, a surface receptor characteristic of an anti-inflammatory activation state, (ii) increased *PCOLCE2* expression, indicative of enhanced extracellular matrix organization, and (iii) elevated ascites levels of IL-6 and IL-10, linked to the aggressiveness of ovarian cancer and immune suppression. In contrast, subgroup B TAMs are characterized by the upregulation of genes linked to immune defense mechanisms and interferon (IFN) signaling. Intriguingly, analysis of published data for 1763 ovarian cancer patients revealed a strong association of this transcriptional signature with a longer overall survival. Consistent with these results, IFNγ was able to abrogate the suppressive effect of ovarian cancer ascites on the inducibility of *IL12B* expression and IL-12 secretion, a key determinant of a cytotoxic immune response.

**Conclusions:**

The survival of ovarian cancer patients is linked to the presence of TAMs with a transcriptional signature that is characterized by a low expression of protumorigenic and immunosuppressive markers and an upregulation of genes linked to interferon signaling. The observed IFNγ-mediated restoration of the inducibility of IL-12 in the presence of ascites provides a possible explanation for the association of an interferon signaling-associated signature with a favorable clinical outcome.

**Electronic supplementary material:**

The online version of this article (doi:10.1186/s12864-017-3630-9) contains supplementary material, which is available to authorized users.

## Background

Ovarian tumors ranks fifth as the cause of cancer-related death in women and represents the deadliest of all gynecological tumors [1, 2]. More than 90% of ovarian cancers are carcinomas that originate from the ovarian surface or fallopian tube epithelium. High grade serous adenocarcinoma is the most common ovarian carcinoma subtype, with most patients presenting with advanced stage disease and disseminated tumor masses at the time of diagnosis. Although most ovarian cancers are highly sensitive to first-line adjuvant chemotherapy, the disease has a dire prognosis with an overall 5-year survival rate of less than 40% [1, 2]. Several characteristic features contribute to the fatal nature of high grade serous ovarian adenocarcinoma, including the shedding of tumor cells at a very early stage of the disease, their spreading via the peritoneal fluid to form transcoelomic metastases and the tumor-promoting and immune suppressive effect of the peritoneal tumor environment, frequently formed by the malignancy-associated effusion within the peritoneal cavity, commonly referred to as malignant ascites. This tumor microenvironment, consisting to a large extent of host-derived cells, is crucial for the growth, progression, therapy resistance and immune escape of malignant tumors, including ovarian cancer [3].

The most common cell types in ovarian HGSC-associated ascites are macrophages and T lymphocytes [3]. Tumor-associated macrophages (TAMs) can be derived from both blood monocytes [4–6] or resident tissue macrophages [7–13], with the latter most likely representing the major origin of TAMs in ovarian cancer [14]. TAM activation is skewed by factors of the tumor microenvironment to adopt a spectrum of phenotypes that represent mixed forms of alternatively activated (immune regulatory) and pro-inflammatory macrophages [15], which has also been clearly demonstrated for TAMs in ovarian cancer ascites [16]. TAMs do not possess tumoricidal activity, but are rather thought to promote immune suppression and various aspects of cancer growth and progression, including tumor cell invasion, angiogenesis and metastasis [15]. Consistent with these tumor-promoting functions of TAMs, expression of the alternative activation marker CD163 in TAMs from malignancy-associated ascites showed a strong correlation with early relapse of serous ovarian carcinoma after first-line therapy [16]. Among the soluble factors contributing to TAM polarization, tumor progression and a poor clinical outcome, interleukin 10 (IL-10), IL-6, transforming growth factor β (TGFβ) and arachidonic acid play a prominent role [14, 16–20].

To date, transcriptional signatures of human TAMs that distinguish subgroups of patients have not been described. In the present study, we address this issue by determining the transcriptome of TAMs from different patients in conjunction with principal component analysis (PCA) and coexpression analysis to define distinct subgroups. These analyses lead to the identification of two subgroups differing in the expression of genes associated with cytokine signaling, immune regulation, extracellular matrix reorganization and overall survival. Of note, an interferon (IFN) related signature showed a striking association with a favorable clinical outcome. Furthermore, IFNγ counteracted repression by ovarian cancer ascites of IL-12, a key mediator of an anti-tumor response mediated by natural killer cells (NK) and T lymphocytes, consistent with the observed association of an IFN signaling-associated signature with ovarian cancer survival.

## Methods

### Patient samples

Ascites was collected from patients undergoing primary surgery at the University Hospital in Marburg. Patient characteristics are presented in Additional file 1: Table S1.

### Isolation and immunophenotyping of cells from ovarian cancer ascites

Mononuclear cells were isolated from ascites by Lymphocyte Separation Medium 1077 (PromoCell) density gradient centrifugation and further purified by magnetic cell sorting (MACS) using CD14 microbeads (Miltenyi Biotech) [16]. TAMs were analyzed by flow cytometry for surface expression of CD14, CD16, CD32, CD64, CD163 and CD206 as described [16]. Tumor cell spheroids and T cells were analyzed as previously published [21].

### Isolation and culture of monocyte-derived macrophages

Monocyte-derived macrophages (MDMs) were generated from monocytes (6-day differentiation period) from healthy donors as described [22] and cultured in RPMI medium with 5% human male AB serum (Sigma-Aldrich, Taufkirchen, Germany) or cell-free ascites from ovarian cancer patients, as indicated. T cell from peripheral blood were isolated as described for ascites [21]. Buffy coats were obtained from the blood bank at UKGM Giessen, Germany.

### RT-qPCR

Isolation of RNA and RT-qPCR were carried out as described [23]. The following primers were used:

- RPL27_fw: 5′AAAGCTGTCATCGTGAAGAAC
- RPL27_rv: 5′GCTGTCACTTTGCGGGGGTAG
- IL12B_fw: 5′GCGAGGTTCTAAGCCATTCG
- IL12B_rev: 5′ACTCCTTGTTGTCCCCTCTG
- CXCL10_fw: 5′AAGCAGTTAGCAAGGAAAGGTC
- CXCL10_rv: 5′GACATATACTCCATGTAGGGAAGTGA
- GBP4_fw: 5′TTCAAAGGCATTAGAGATTCTTGA
- GBP4_rv: 5′GTGGAGCCCAGAGGGAAG
- GPNMB_fw: 5′CTATGAGAAGAACTGCAGAAATG
- GPNMB_rv: 5′GTTATGATGGCTTTGGCCGG
- LGALS2_fw: 5′CCACGAGTTGAGCCCTGAG
- LGALS2_rv: 5′CGGCTTCATGTCCATGTTC
- KITLG_fw: 5′GCCAAGTCTTACAAGGGCAG
- KITLG_rv: 5′GAAACTCTCTCTCTTTCTCTTGC
- MRC1_fw: 5′CCT CGG ACC TGG TTA GGG
- MRC1_rv: 5′GGATGTGTGGTCCTCCTTGG

### ELISA

Concentrations of p40 (IL-12B/IL-23) in cell culture supernatants were determined using an ELISA Kit from BioLegend/Biozol (Eching, Germany) according to the instructions of the manufacturer. IFNγ, IL-6 and IL-10 levels in ascites were quantified by ELISA kits purchased from eBioscience (Frankfurt, Germany).

### RNA sequencing (RNA-Seq)

RNA isolation and RNA-Seq was carried out on an Illumina HiSeq 1500 as described [21]. Genome assembly and gene model data were retrieved from Ensembl revision 81. Sequencing data were deposited at EBI ArrayExpress (accession numbers E-MTAB-5199 and E-MTAB-4162).

### Statistical analysis of experimental data

Paired and unpaired t tests were carried out with the Python functions *scipy.stats.ttest_rel ()* and *scipy.stats.ttest_ind* (), respectively. Results were expressed as follows: *p < 0.05; **p < 0.01; ***p < 0.001; ****p < 0.0001. Confidence intervals were calculated using the bootstrap method. Further statistical analyses were performed using the Python functions *numpy.percentile () and pandas.DataFrame.boxplot ().*

### Analysis of RNA-Seq data

RNA-Seq data was aligned to Ensembl v81 using STAR (version 2.3.1z13_r470) and processed as reported [21]. The number of mapped reads was in the range of 19.74–35.92 million (median 29.42 million). TPM (transcripts per million) values were calculated based on the total gene read counts and length of merged exons and corrected for contamination by tumor cells as described [21]. The source code for implementing the algorithm for TPM correction has also been deposited at GitHub (https://github.com/IMTMarburg/rnaseqmixture). Genes were considered expressed if they had a minimum TPM value of 3. TAM samples with TPM >50 for *EPCAM* or *MSLN* were excluded due to presumed tumor cell contamination. All genomic sequence and gene annotation data were retrieved from Ensembl release 81, genome assembly hg38.

### PCA and delineation of differentially expressed gene clusters

PCA was carried out on using *the sklearnPCA(n_components = 2, whiten = True)* and *sklearn_pca.fit_transform ()* functions (Python) on RNA-Seq data. Pearson correlation coefficients (r) were determined with *scipy.stats.pearsonr ()*. The Bioconductor package edgeR [24] was used for the delineation of high variance gene clusters differentially regulated in subgroups of TAMs identified by PCA (signatures 1 and 2).

### Coexpression analysis

Genes with the greatest expression variance were identified by *pandas.DataFrame.var ()*. Pearson correlation coefficients (r) were determined for the 3000 top genes using *scipy.stats.pearsonr ()*. Sets of genes with r > 0.89 and *n* > 10 were combined (*n* = 629) and analyzed by hierarchical clustering using the *scipy.cluster.hierarchy* functions *linkage (method = “weighted”, metric = “correlation”)* and *dendrogram (truncate_mode = “none”, color_threshold = 0.8)*. The resulting 4 clusters (I, II, III, IV) were analyzed for intersections the signatures identified by PCA (see above), which revealed close relationships of cluster I with signature B and cluster III with signature A.

### Pathway analyses

Gene sets were analyzed for *Upstream Regulators* using the Ingenuity Pathway Analysis (IPA) database (Qiagen Redwood City, CA, USA) as described [22]. Functional annotations were performed by gene ontology (GO) enrichment analysis (http://geneontology.org).

### Survival analyses

Overall survival (OS) data were retrieved from PRECOG (https://precog.stanford.edu) [25]. Associations between gene expression and relapse-free survival (RFS) were analyzed by the web-based tool “KM Plotter” [26] (http://kmplot.com) using the following settings: ‘auto select best cutoff’, probe set option: ‘JetSet best probe’, histology: serous, datasets: all; other settings: default.

## Results

### Clustering of ovarian carcinoma TAM samples

**Fig. 1:**
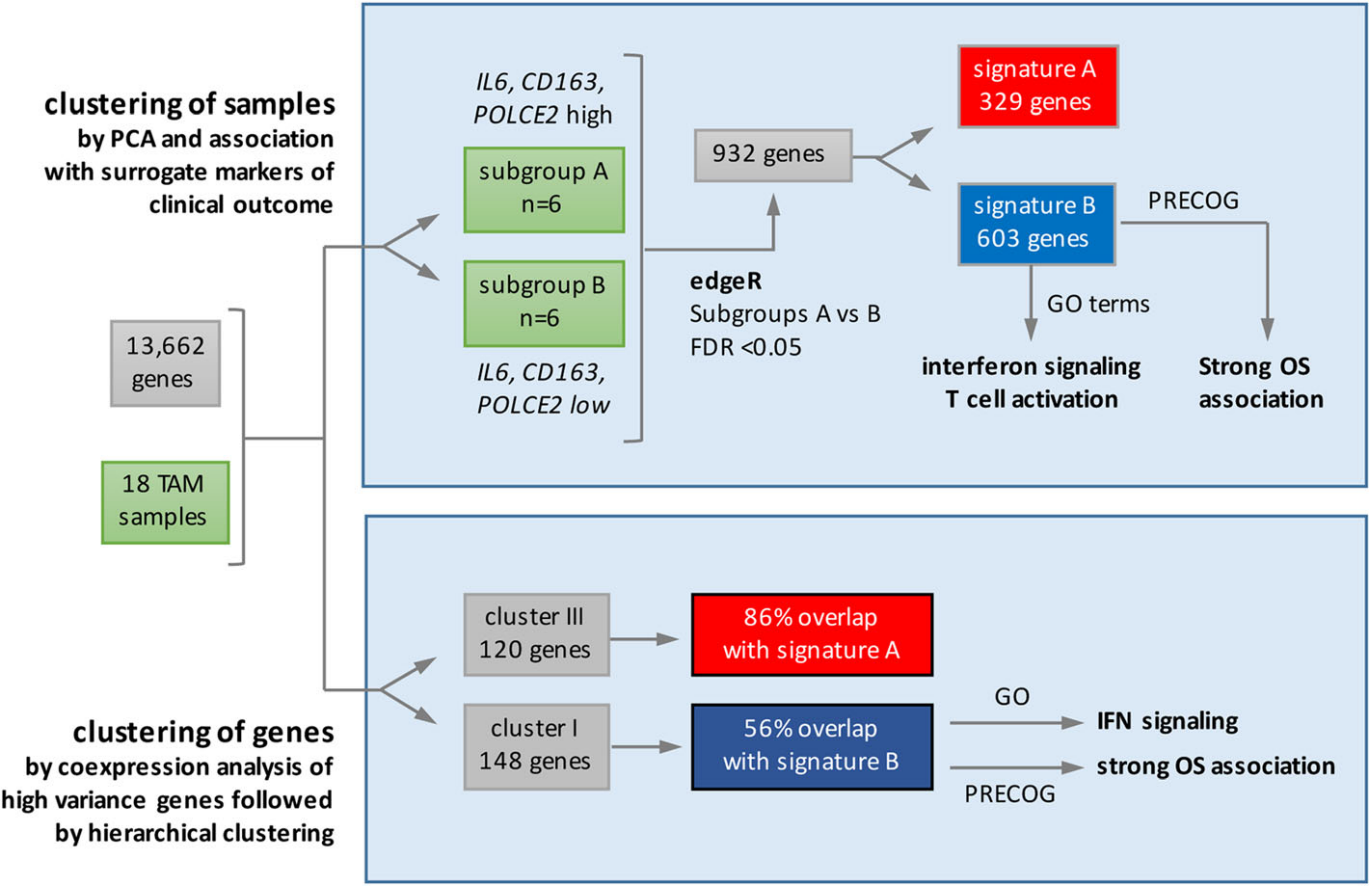
Schematic representation of data analysis and summary of results. Nomenclature for designation of clusters: TAM samples clustered by PCA: letters (A, B); genes clustered by coexpression analysis: roman numbers (I, II, III). Genes identified by edgeR and upregulated in TAM subgroup A or B were defined as signature A and B, respectively

The transcriptomes determined for 18 independent samples of TAM isolated from the ascites of ovarian cancer patients (Additional file 2: Dataset S1) were analyzed for potential similarity patterns by different approaches, as schematically summarized in Fig. 1. These samples were selected for very low contamination with tumor cells, as indicated by low TPM values (<50) for the epithelial marker genes *EPCAM* and *MSLN*. Furthermore, we excluded all genes (*n* = 13) highly expressed in tumor cells or T cells versus TAMs (>100-fold) to minimize interference by contaminating cells.

**Fig. 2:**
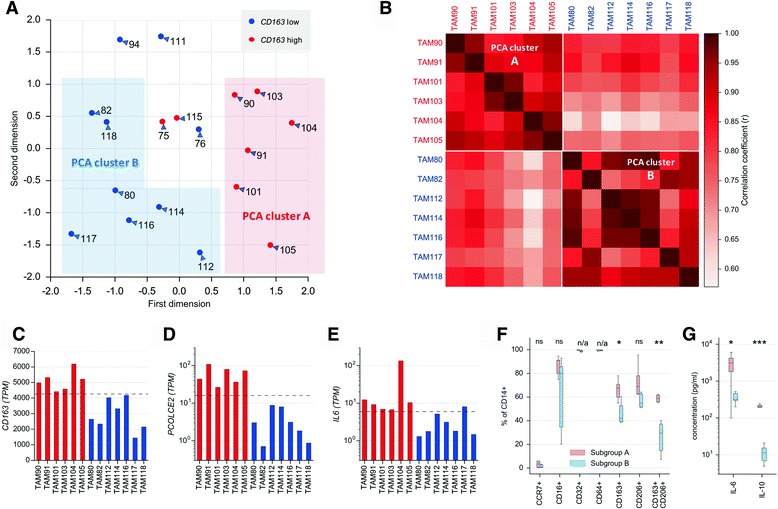
Clustering of ovarian carcinoma TAM samples based on RNA-Seq data. **a** Principal component analysis (PCA) of TAM transcriptomes. Samples with high expression of *CD163* (TPM > median) are shown in *red* (sub A), samples with low expression of *CD163* in *blue* (sub B). **b** Heatmap based on Pearson correlation coefficients (r) calculated for the TAM transcriptomes identified by PCA (sorted by subgroups). **c-e** Expression of *IL6, PCOLCE2* and *CD163* in TAM samples of clusters A (*red*) and B (*blue*). *Dotted lines* show the quantiles used in panel (**a**). **f** Flow cytometry analysis of cluster A and B samples. The *plot* shows the fraction of CD16^+^, CD32^+^, CD64^+^, CD163^+^, CD206^+^ and CD163^+^CD206^+^ cells (of CD14+ cells). **g** Concentrations of IL-6 and IL10 in the ascites of cluster A and B patients determined by ELISA. *Boxes* show the upper and lower quartiles, whiskers the 95% confidence intervals (CI), and horizontal lines the median. *Asterisks* indicate the statistical significance determined by unpaired *t* test (cluster A versus B samples). n/a, not applicable since all values >97%; ns: not significant

Genewise normalized TAM transcriptomes were used for the bioinformatic delineation of similarity patterns. Principal component analysis (PCA) did not yield a clear partitioning of TAM samples into subgroups, although eigenvalues suggested that the first two components can explain most of the data. We therefore additionally grouped samples according to the expression of *CD163*, previously established as a TAM marker associated with a poor clinical outcome of ovarian cancer [16]. Combining PCA with the the expression pattern of this surrogate marker revealed two discernible subgroups (A and B; Fig. 2a), which was confirmed by Pearson correlation (Fig. 2b) and distance-based multidimensional scaling analysis (Additional file 3: Figure S1). Two other markers associated with pro-tumorigenic functions, i.e., *IL6* (interleukin 6) [14, 16–21, 27] and *PCOLCE2* (procollagen C-endopeptidase enhancer 2; see Additional file 3: Figure S2), showed a very similar pattern of expression, except for *IL6* expression in TAM117 (Fig. 2c-e). Based on this data we defined subgroup A as TAM90, TAM91, TAM101, TAM103, TAM104 and TAM105 expressing *CD163, PCOLCE2* and *IL6* at high levels relative to the subgroup B samples TAM80, TAM82, TAM112, TAM114, TAM116 and TAM118. These subgroups were confirmed by flow cytometry showing a significantly higher fraction of CD163^+^ and CD163^+^CD206^+^ cells in subgroup A versus subgroup B TAMs, which was not observed for other macrophage markers (Fig. 2f).

Taken together, these data indicate that cluster A TAMs are skewed towards alternative activation (CD163), extracellular matrix (ECM) remodeling (PCOLCE2) and promotion of tumor growth (IL-6). As these markers are associated with a short relapse-free survival, it is likely that cluster B is linked to a favorable clinical outcome. This conclusion is supported by the observation that the ascites concentrations of IL-10, highly predictive of a poor survival of ovarian cancer [21, 28] was consistently and dramatically increased in subgroup A versus subgroup B patients (Fig. 2g). A similar pattern was observed with IL-6 (with one outlier; Fig. 2g), also associated with a short time to relapse [17, 18, 21].

### Cluster-specific gene expression

**Fig. 3:**
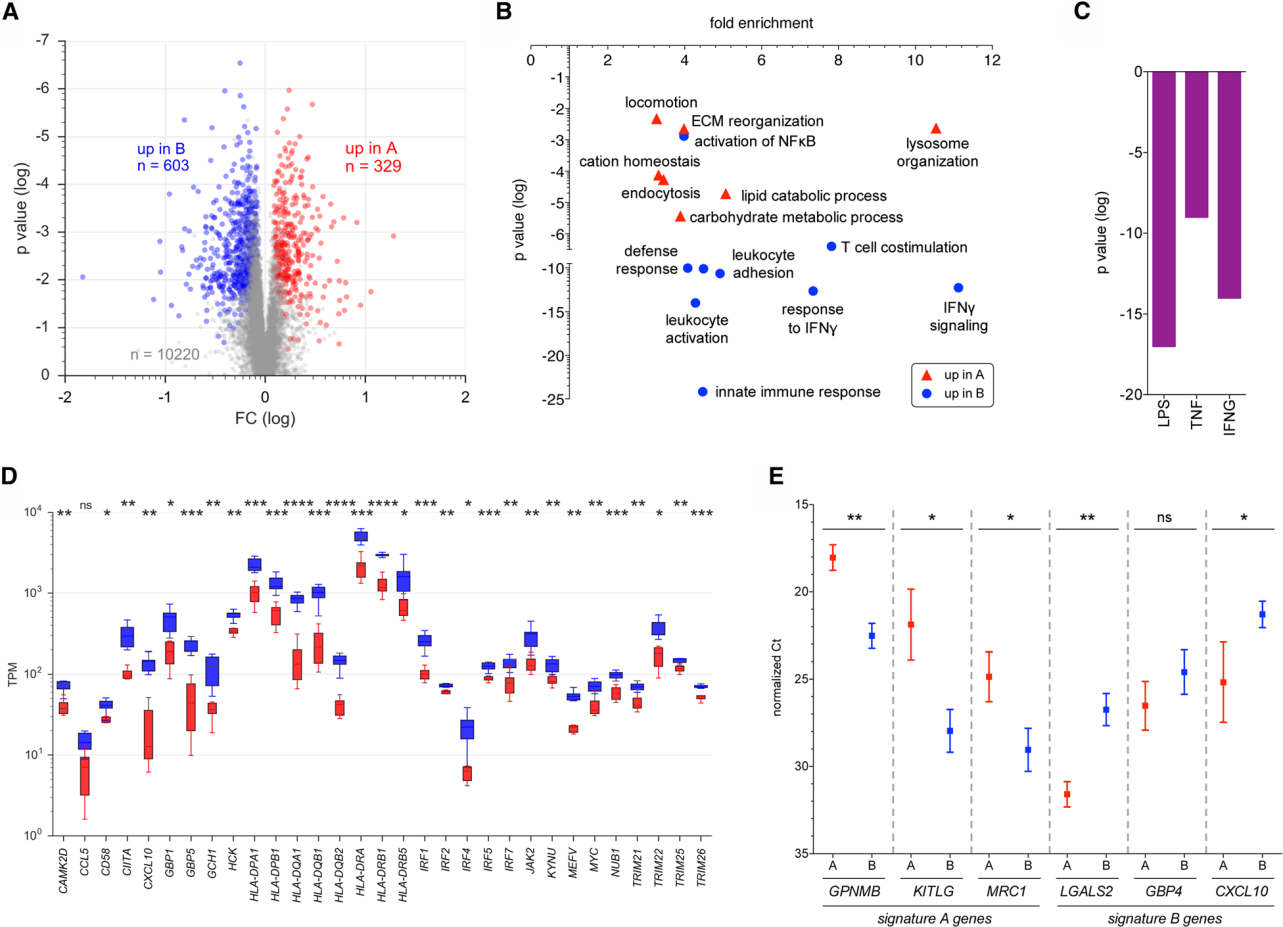
Identification of differentially expressed genes in subgroup A versus B TAMs by edgeR. **a** *Scatter plot* showing the expression of genes identified by the edgeR tool (FDR <0.05) in TAM subgroups A or B identified by PCA in Fig. 2. Data represent the ratio (FC) of median TPM values for subgroup A versus subgroup B. **b** Functional annotation of genes upregulated in subgroup A (*red*) or subgroup B (*blue*) by gene ontology (GO) enrichment analysis. *p* values are plotted against fold enrichment. Only specific non-redundant terms with *p* values <0.01 and enrichment >3 are shown. **c** Upstream Regulator Analysis (Ingenuity Pathways Analysis database) of upregulated genes with *p* < 10^−8^. **d** Expression of the IFN signaling-associated genes of signature B identified by GO enrichment analysis (**c**). *Boxes* show the upper and lower quartiles, whiskers the 95% CI, and *horizontal lines* the median. **e** Validation of RNA-Seq data. Analysis by RT-qPCR of signature A and B genes (Additional file 2: Datasets S3 and S4) in TAM samples from subgroup A and B (*n* = 6). *Error bars* show the standard deviation and *horizontal lines* the mean. *Red*: cluster A samples; *blue*: cluster B samples. *Asterisks* indicate the statistical significance determined by unpaired *t* test (cluster A versus B samples); ns: not significant

To gain more insight into the specific phenotypes of subgroup A and B TAMs, we analyzed the RNA-Seq data sets with edgeR, a Bioconductor package for reliable gene-specific dispersion estimation in small datasets [24]. The edgeR tool identified a group of 932 genes differentially expressed in the two subgroups of TAMs with an FDR of <0.05 (Additional file 1: Table S1; Fig. 3a; Additional file 2: Dataset S2). Of these, 329 genes were upregulated in subgroup A versus B, and 603 genes showed the opposite pattern (Fig. 3a; Additional file 2: Datasets S3 and S4). These gene sets were termed signature A and signature B. In contrast to these protein coding RNAs, edgeR did not identify any differentially expressed lncRNAs (FDR <0.05) in our RNA-Seq data set (annotated lncRNAs: *n* = 7527).

Gene ontology (GO) enrichment analysis identified significant associations of signature A with diverse biological processes, including ECM remodeling, locomotion, endocytosis as well as lipid and carbohydrate catabolism (Fig. 3b). In contrast, innate immune defense mechanisms, T cell activation and IFN signaling were strongly associated with signature B (Fig. 3b). Consistent with these findings, Ingenuity Upstream Regulator Analysis indicated that signature B genes are major targets of proinflammatory pathways triggered by lipopolysaccharide (LPS), tumor necrosis factor α (TNF) and INFγ (Fig. 3c), with all three target gene sets showing strong overlaps (Additional file 3: Figure S3).

The differential expression patterns of signature A and B genes are shown in Fig. 3d and Additional file 3: Figure S4 for the IFN and ECM gene sets identified by GO enrichment analysis as representative examples. As expected, these gene sets showed opposite patterns of regulation and the subgroup-selective expression was clearly significant for the vast majority of genes (*n* = 48 out of *n* = 51). The RNA-Seq data for several genes identified by edgeR were confirmed by RT-qPCR (Fig. 3e).

Taken together, these observations point to a relatively high complexity of biological functions affected by signature A genes, while the role of signature B genes appears to be specifically associated with IFN-stimulated immune defense mechanisms.

### Confirmation of clustering by coexpression analysis

**Fig. 4:**
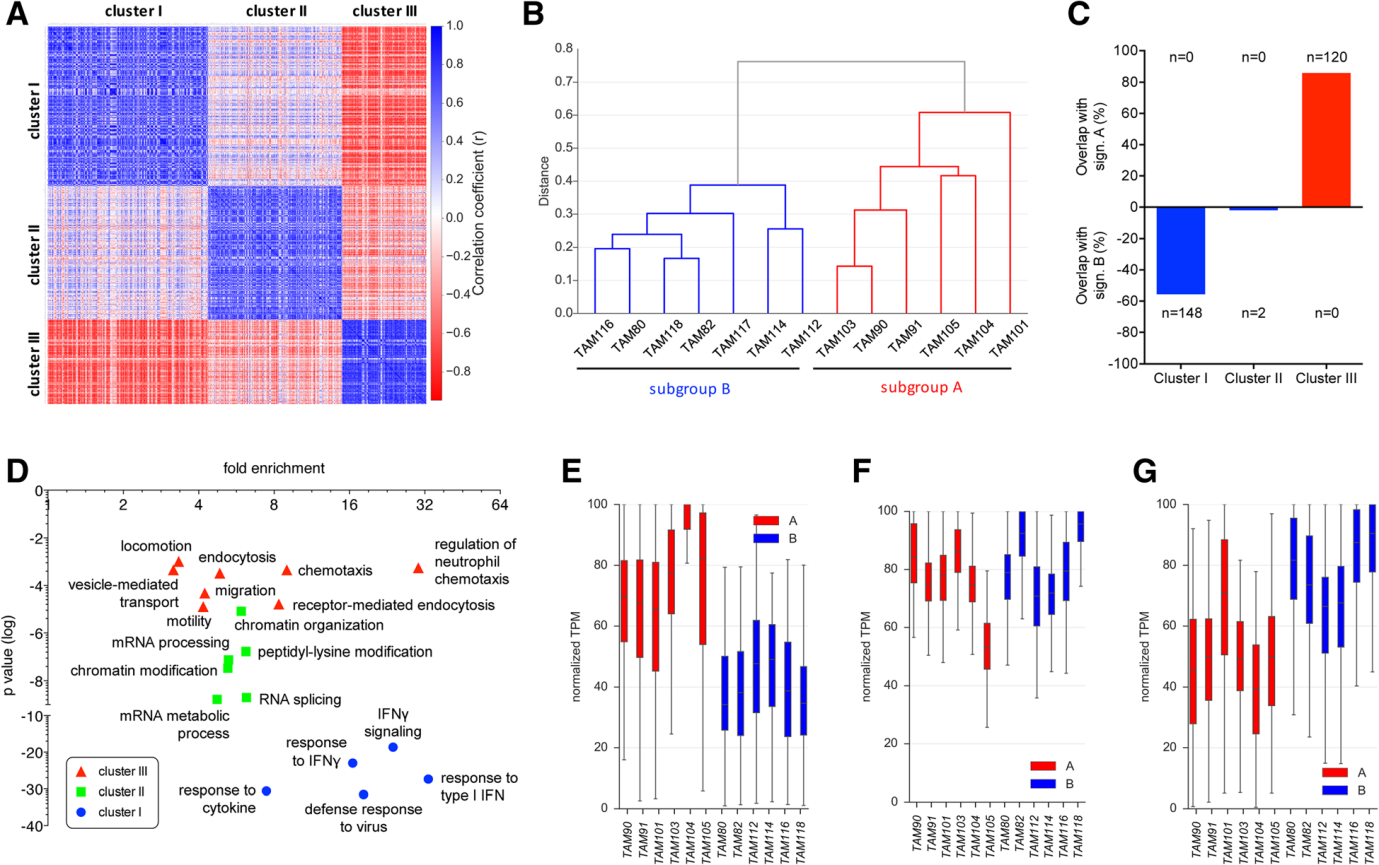
Coexpression analysis of all TAM samples. **a** Correlation based heatmap of gene clusters (I, II and III; 265, 222 and 139 genes, respectively) defined by coexpression analysis of genes with the highest variance across all TAM samples, followed by hierarchical clustering (see Additional file 3: Figure S4 for a dendrogram). **b** Hierarchical clustering of patients based on the genes identified in panel (**a**). **c** Overlap of genes in clusters I - IV with signatures A and B identified by PCA. **d** Functional annotation of cluster I (*blue*), cluster II (*green*) and cluster III (*red*) genes by gene ontology (GO) enrichment analysis. *p* values are plotted against fold enrichment. Only specific non-redundant terms with *p* values <0.0001 and enrichment >3 are shown. **e** Expression of cluster I genes (*n* = 120; genewise normalized TPM values) in subgroup A (*red*) and B (*blue*) samples. **f** As panel (**e**), but for cluster II genes (*n* = 223). **g** As panel (**e**), but for cluster III genes (*n* = 148). *Boxes* show the upper and lower quartiles, whiskers the 95% confidence intervals (CI), and *horizontal lines* the median

To obtain independent evidence for the robustness of the clusters defined by PCA and edgeR we performed coexpression analysis the genes showing the highest variance across all TAM samples. Pearson correlation and hierarchical clustering yielded three large clusters of coregulated genes (Fig. 4a; Additional file 3: Figure S5; Additional file 2: Datasets S5-S7). Hierarchical clustering of TAM samples using a combined set of these genes (Fig.  4a) yielded the identical partitioning into subgroups A and B as the PCA-based approach in Fig. 2a. Consistent with this observation, two of these gene clusters showed substantial and specific overlaps with signatures A and B, respectively, i.e., cluster I with signature A (120/140 = 85,7% and no overlap with signature B); cluster III with signature B (148/266 = 55.6% and no overlap with signature A), as shown in Fig. 4c as well as Additional file 2: Datasets S8 and S9.

Functional annotations revealed endocytosis and chemotaxis for cluster I, chromatin modification and splicing for cluster II, and immune defense and interferon signaling for cluster III (Fig. 4d). Consistent with the overlaps of described in the preceding paragraph, the GO terms for cluster I were also found for signature B, and the terms for cluster III correspond to those for signature A (compare Figs. 3b and 4d).

Finally, expression of cluster I genes was higher in subgroup B versus subgroup A (Fig. 4e), and vice versa, expression of cluster III genes was higher in subgroup A relative to subgroup B (with the exception of TAM101 (Fig. 4g). In contrast, no differential expression was observed for cluster II genes (Fig. 4f). Taken together, these findings clearly suggest that cluster III corresponds to signature A, while cluster I corresponds to signature B. Thus, both strategies, PCA of patient samples followed by edgeR and coexpression analysis of high variance genes yielded very similar results, and identified IFN signaling as a hallmark of signature B upregulated in subtype B TAMs.

### Association of signature B, interferon signaling and survival

In order to identify potential associations between the expression of this genes in ovarian cancer with clinical outcome we made use of the PRECOG database, which contains the results of a meta-analysis of 1763 patients from 12 studies [25]. These studies used solid ovarian tumor tissue containing substantial amounts of myeloid cells [25, 29] for transcriptome analysis, suggesting that the PRECOG data are also suitable for testing survival associations for genes expressed in abundant tumor-associated host cells, such as TAMs.

**Fig. 5:**
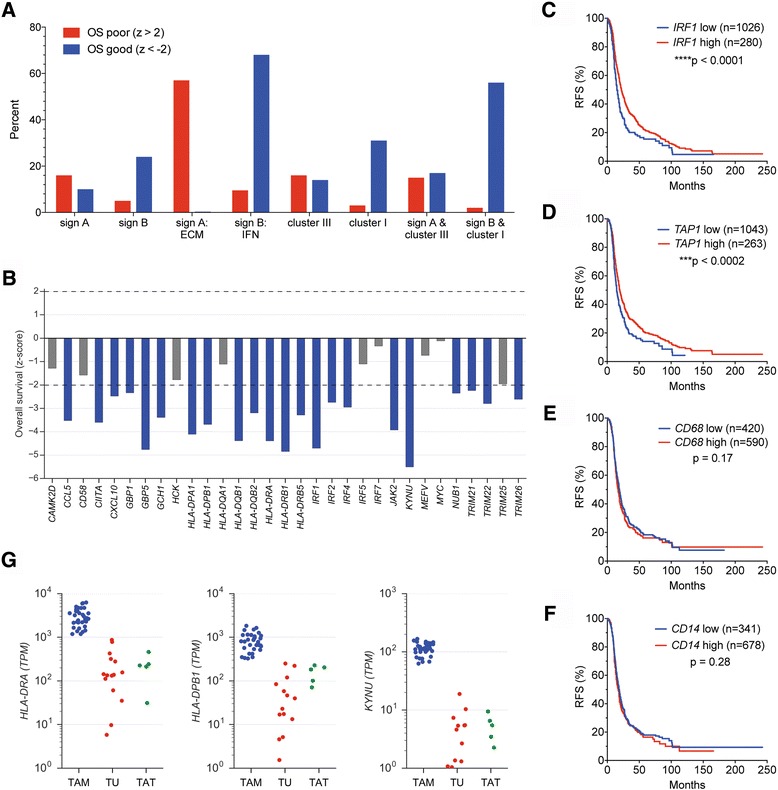
Association of cluster-specific gene expression with ovarian cancer survival. **a** Mean z-scores (OS) for signature A and B genes; the ECM-related and IFN signaling-associated genes of signature A and B, respectively; cluster III and I genes; and genes representing the intersection of signature A with cluster III or of signature B with cluster I. Survival data were obtained from the PRECOG database with 1763 ovarian cancer patients [25]. **b** OS z-scores for signature B genes that are associated with IFN signaling. Significant associations with a favorable clinical outcome are shown in *blue* (z-score < −2.0; HR <1). The corresponding data for the complete signatures A and B are shown in Additional file 3: Figure S6. **c**-**f** Kaplan-Meier plots analyzing the association of *IRF1*, *TAP1, CD14* and *CD68* with RFS of high-grade serous ovarian cancer determined by KM plotter [26]. **g** Expression of the signature B genes *HLA-DPB1, HLA-DRA* and *KYNU* in TAMs (*blue*, *n* = 33), tumor cells (*red*, *n* = 15) and CD3^+^ TATs (*green*, *n* = 5) isolated from ovarian cancer ascites

Signature A, cluster III and their overlap comprised similar fractions of genes associated with poor or favorable OS (Fig. 5a and top panel in Additional file 3: Figure S6). Only when genes with distinct functional annotations were analyzed separately a clear OS association was detectable. Thus, signature A genes linked to ECM remodeling were strongly associated with a short OS (Fig. 5a and Additional file 3: Figure S7). In contrast, signature B, cluster I and their overlap were clearly linked to a favorable clinical outcome (Fig. 5a and bottom panel in Additional file 3: Figure S6). This applied in particular to the IFN signaling-associated genes of signature B, as shown in detail in Fig. 5b. A similar inverse association with relapse-free survival was also found based on data from the KM plotter database, as exemplified for IRF1 and TAP1 in Fig. 5c and d. All PRECOG z-scores for signature A and B genes are also listed in Additional file 2: Dataset S10.

These associations with a favorable clinical course are not simply consequence of the extent of myeloid cell infiltration. First, activation state-independent myeloid marker genes, such as *CD14* or *CD68* did not show any significant association with ovarian cancer OS (PRECOG) [25] or RFS [26] (Fig. 5e and f). Second, tumor infiltration by myeloid cells inferred from RNA expression data (CIBERSORT) [29] showed no significant association with OS, with a trend towards a worse clinical outcome for monocytes (Additional file 3: Figure S8). Third, a number of the IFN signaling-associated genes of signature B are expressed at high levels (TPM > 10) selectively in TAMs compared to tumor cells or tumor-associated CD3^+^ lymphocytes (TATs) isolated from ovarian cancer ascites, as exemplified by *HLA-DRA, HLA-DPB1* and *KYNU* in Fig. 5g).

### Expression of IFN-encoding genes is linked to ovarian cancer survival

**Fig. 6:**
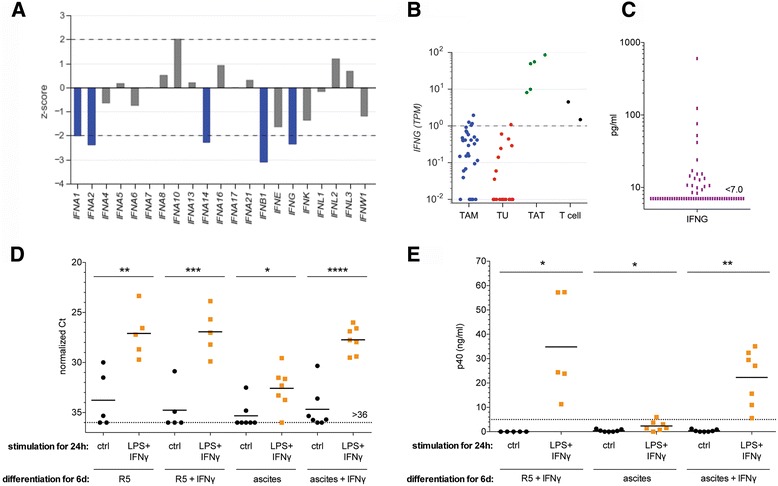
Associations of *IFN* gene expression with survival and abrogation by IFNγ of the ascites-induced *IL12B* activation block. **a** z-scores for the association of *IFN* genes with OS (PRECOG data). *Blue bars*: significant associations with a favorable clinical outcome (z-score < −2.0; HR <1). **b** Expression of *IFNG* in TAMs (*n* = 33), tumor cell (*n* = 22) and TATs (*n* = 5) samples from ovarian carcinoma ascites, and in CD3^+^ T cells from healthy donors (*n* = 2). Each *dot* represents an individual sample (see Additional file 2: Dataset S1 for details). **c** IFNγ concentrations in the ascites from *n* = 61 ovarian cancer patients determined by ELISA. **d** *IL12B* expression in MDMs differentiated for 6 d either in RPMI plus 5% human A/B serum (R5 medium) or in ovarian cancer ascites in the absence or presence of IFNγ (50 ng/ml). Cultures were stimulated with LPS (100 ng/ml) plus IFNγ (20 ng/ml) or solvent only (Ctrl) for 24 h and RNA was analyzed by RT-qPCR. The experiment was performed with 7 independent samples (combinations of 5 donors and 5 ascites samples). **e** p40 (IL-12B/IL-23) protein concentrations in the culture medium of the experiments in panel (**d**). Each *dot* represents an independent sample. *Horizontal lines*: median. Significance was determined by *t*-test between unstimulated and IFNγ + LPS-stimulated cells

Analysis of the PRECOG database also revealed a significant association of *IFNG* expression with a longer survival (z < −2; Fig. 6a). Similar associations were also found for several genes coding for type I IFNs, i.e., *IFNA1, IFNA2, IFNA14* and *IFNB1*. Evaluation of RNA-Seq data for different cell types [21] (Additional file 2: Dataset S1) showed that tumor-associated T cells (TATs) express *IFNG* at relatively high levels (TPM = 10–100), while TAMs and tumor cells do not (Fig. 6b). Interestingly, all TAT samples expressed *IFNG* at higher levels than normal CD3^+^ T cells isolated from the blood of healthy donors, with >10-fold higher levels observed with three out of the five patients analyzed. It is therefore likely, that partially activated TATs are a major source of IFNγ within the tumor microenvironment and the malignancy-associated ascites. This is consistent with the presence of readily detectable IFNγ levels in the ascites of a subgroup of patients (*n* = 21 above ELISA detection limit out of a total of *n* = 61 samples; Fig. 6c). By contrast, all type I IFN genes associated with a favorable OS were expressed a very low levels by all three cell types, if at all (Additional file 3: Figure S9).

### IFNγ prevents the ascites-induced *IL12B* activation block in macrophages

Interleukin-12 (IL-12) is a particularly interesting cytokine in the context of ovarian cancer due to its immune stimulatory anti-tumor effects and its inverse associations with disease progression patients [30–32]. A hallmark of TAMs in ovarian cancer ascites is their defect to release IL-12 in response to inflammatory stimuli, which results from a transcriptional block of the *IL12B* gene encoding the p40 subunit [14, 33, 34]. Another cytokine with beneficial immune stimulatory and anti-tumor effects in ovarian cancer patients is IFNγ [35–38], consistent with the observation that IFNγ can prevent the skewing of monocyte differentiation by ovarian cancer ascites from immunostimulatory IL-10^low^IL-12^high^ macrophages to TAM-like IL-10^high^IL-12^low^ cells [34]. To assess the relevance of the IFN signaling-associated signature B we explored the relationship between IL-12 and IFNγ in further detail.

Toward this end, we measured the inducibility of *IL12B* mRNA by lipopolysaccharide (LPS) plus IFNγ in monocyte-derived macrophages that were differentiated either in the presence of regular cell culture medium (R5) or in ascites, both in the absence or presence of IFNγ during macrophage differentiation. As expected, all ascites samples tested blocked *IL12B* induction, which, however, was almost completely prevented by IFNγ (Fig. 6d). In full agreement with these findings, secretion of p40 was strongly induced by LPS plus FNγ in R5, which was blocked by ascites only in the absence of IFNγ during differentiation (Fig. 6e). These results clearly point to a clinically beneficial IFNγ – p40/IL12B axis in differentiating myeloid cells in the ovarian cancer microenvironment, thus providing a potential explanation for the association of subgroup B TAMs with clinically favorable parameters.

## Discussion

### Delineation of subgroups of TAMs

Transcriptional signatures of TAMs distinguishing subgroups of patients in a biologically or clinically meaningful way have not been reported to date. We have used the ovarian cancer-associated ascites as an experimental system to address this issue by an unbiased approach (Fig. 1). By applying PCA to data derived by next generation sequencing we were able to split TAM samples from different patients into subgroups characterized by distinct gene expression patterns (Fig. 2). Remarkably, cluster B is basically congruent with the subgroup of patients with low expression of the *IL6, PCOLCE2* and *CD163* genes, a low fraction of anti-inflammatory CD163^+^/CD206^+^ TAMs and low ascites concentrations of IL-10 and IL-6 (Fig. 2c-g). These features are known as negative prognostic factors for different tumor entities, including ovarian cancer [16–18, 21, 28, 39, 40]. The same subgroups were also identified by an another unbiased approach, i.e., coexpression analysis of all TAM samples (Fig. 4). Therefore, these findings clearly suggest that subgroups A and B represent patients with a poor and favorable clinical outcome, respectively.

### IFN signaling is a hallmark of signature B TAMs

Intriguingly, a hallmark of subgroup B is the upregulation of target genes of the IFN signaling network (Figs. 3b and 4d). Consistent with this finding, our analysis of the PRECOG database revealed a strong association of these genes with a longer OS (Fig. 5b). This is in agreement with previous studies which associated a high protein expression of several IFN signaling components with a favorable clinical outcome, including IRF1 [41]. Furthermore, IFNγ inclusion in the first-line therapy of ovarian cancer resulted in an effector immune cell response [35] and a prolongation of progression-free survival [36, 37], while type I IFNs had no benefit [42]. In keeping with these observations, elevated *IFNG* expression in ovarian cancer tumor tissue correlated with an improved clinical outcome in patients [43].

Our upstream regulator and functional annotations (Figs. 3b, c and 4c) yielded IFNγ and type I IFN as activated signaling pathways. We attribute this apparent ambiguity to the fact that type I and II target genes show substantial overlaps. Type I IFNs signal through their common receptor complex via JAK1/TYK2 to the heterotrimer ISGF3 (STAT1:STAT2:IRF9) and, to a lesser extent, through STAT1 homodimers, whereas IFNγ, the single type II IFN, uses only STAT1 homodimers phosphorylated by IFNγ receptor-associated JAK1/JAK2 [44]. Furthermore, STAT-independent pathways could be activated differentially by the different receptor complexes. Taken together, this explains the overlapping, but not identical, effects of the two types of IFNs. Interestingly, *JAK2* is among the signature B genes identified in this study (Fig. 3d, Additional file 2: Dataset S4) and might thus contribute to IFNγ-mediated effects. Taken together with our observation that, in contrast to *IFNG* (Fig. 6b), none of the type I IFN genes associated with a longer OS is expressed at significant levels by TAMs, TATs or tumor cells (Additional file 3: Figure S9) it is conceivable that upregulation of the IFN target genes of signature B is due to activated IFNγ signaling.

Collectively, these findings suggest that the increased expression of IFN target genes in cluster B TAMs results, at least in part, from an elevated level of IFNγ in the tumor microenvironment. Of note, a substantial fraction of the IFN signature genes upregulated in cluster B TAMs are also target genes of pro-inflammatory pathways (Additional file 3: Figure S3). This suggests that other cytokines present in ascites, notably TNFα [45], might contribute to the induction of these genes. This would be consistent with the prevailing opinion that pro-inflammatory macrophages inhibit tumor progression.

Since RNA-Seq measures mean transcript levels within a cell population, the fraction of TAMs expressing a given signature cannot be determined. It is therefore possible that elevated expression of IFN genes reflects the higher relative occurrence of a subpopulation of cells. These might be a newly recruited, CD163-negative, monocyte-like TAM subset similar to the macrophage fraction that is able to elicit inflammatory responses in immune privileged reproductive organs [46]. This hypothesis is in agreement with the flow cytometry data in Fig. 2f, indicating that CD163^+^ and CD163^−^ cells occur in both subgroup A and B, even though their ratio is clearly different in both subpopulations. Likewise, it is possible that a few TAM samples could not be fitted into either subgroup (Fig. 2a) due to the presence of similar fractions of functionally different subpopulations.

### Role of T cells

Ascites contains substantial numbers of different types of T cells, in particular CD4^+^ and CD8^+^ cells [47, 48], known as important IFNγ producers under physiological conditions. A functional role of T cells in ovarian cancer is supported by many published observations strongly associating infiltrating T cells with a longer survival, with a high ratio of CD8^+^ versus regulatory T cells having a strong impact [47–49]. Consistent with these findings, transcriptome analyses defined distinct high-grade serous ovarian carcinoma subtypes, of which the immune reactive subtype was associated with the best prognosis [50]. Transcriptome profiling also identified several genes contributing to cytotoxic T lymphocyte recruitment as being differentially expressed in tumors with high versus low CD8 T cell infiltration, including IRF1 [51–53], providing another link between T cells, IFNγ and a favorable clinical outcome. Furthermore, the analysis in Fig. 6a revealed a positive impact of *IFNG* expression on ovarian cancer OS. It is thus possible that IFNγ in the tumor microenvironment and the expression of IFN target genes in TAMs are indicators of the presence of activated, IFNγ secreting T and/or NK cells mediating anti-tumor immune responses.

Our RNA-Seq data support the conclusion that TATs are a major source of IFNγ in ascites (Fig. 6b). Since TATs showed a considerably higher level of *IFNG* expression compared to normal T cells, it is likely that the former are partially activated, at least in a subset of patients. This would be consistent with the observed clonal expansion of T cell subpopulations of unknown biological relevance in ovarian cancer ascites [54]. However, TATs apparently are not functional with respect to an anti-tumor response, as suggested by progression of the disease, presumably due the inhibitory effect of ascites on T cell activation [55]. In line with this conclusion other makers of an activated TH1, TH2, TH9 or TH17 response were only weakly upregulated in a subset of TATs relative to normal T cells (e.g., *FASLG, GZMA, TNF*), expressed at similar levels in both (e.g., *CCR4, IL10, LAMP1/CD107A, LTA, PRF1*) or not expressed at all (e.g., *IL2, IL4, IL9, IL12B, IL13, IL17A*) (Additional file 2: Dataset S1).

### Inflammatory signaling and IL-12 induction

There is a large body of evidence to suggest that IFNγ and IL-12 are key determinants of a beneficial immune response in many cancers [56]. Physiologically, IL-12 is released by macrophages and other antigen-presenting cells in response to proinflammatory signals, including toll-like receptor ligands and IFNγ from T or NK cells. IL-12 in turn stimulates a cytotoxic response by inducing multiple immune stimulatory processes, including the differentiation of naive T cells into Th1 cells, the activation of CD8 + T cells and the maturation or activation of NK cells [57].

Multiple observations strongly support the hypothesis that IL-12, and probably its induction by IFNγ, are crucial determinants of ovarian cancer outcome. For example, IL-12 locally produced significantly delayed peritoneal disease development in a mouse model [32], engineered tumor-targeted T cells ectopically expressing a fused *IL12A/IL12B* cDNA have been reported to eradicate ovarian tumors in vivo [31] and a highly significant association was found between high *IFNG* and *IL12B* expression and a more favorable clinical outcome of advanced stage ovarian carcinoma [30].

In TAMs, the acquisition of a proinflammatory phenotype, including the secretion of IL-12, in response to proinflammatory signals is non-functional. In LPS-stimulated murine macrophages, *Il12b* induction is selectively dependent on the NFκB family member Rel [58] and on TLR signaling-induced chromatin remodeling which is independent of Rel [59]. Furthermore, IFNγ has been shown to enhance the synthesis of IL-12 by priming macrophages for LPS-mediated induction of the *IL12B* gene [60]. Finally, the nuclear accumulation of a NFκB p50 homodimer with presumed inhibitory function has been suggested for the acquisition of a TAM phenotype characterized by the defective production of IL-12 [33]. Intriguingly, IFNγ can prevent the inhibitory effect of ascites on the inducibility of *IL12B* in macrophages (Fig. 6d, e), consistent with a previous report that IFNγ was able to shift monocyte differentiation from TAM-like cells to pro-inflammatory macrophages [34]. It is currently unclear if, and if so how, TLR pathways, IFNγ triggered STAT signaling, chromatin remodeling and p50 accumulation functionally interact in the regulation/dysregulation of the *IL12B* gene in either normal macrophages or TAMs. Understand these connections will be crucial to be able to explore the potential of the IFNγ – IL-12 axis in stimulating cytotoxic immune responses and assess potential applications.

## Conclusions

In the present study, we have address the question as to whether associated-derived TAMs from different patients with ovarian cancer represent a continuum of overlapping transcriptomes or can be categorized into phenotypically distinct groups on the basis of their global gene expression patterns. The results of both principal component analysis (PCA) and coexpression analysis clearly demonstrated that the latter is the case, and lead to the definition of two highly distinct subgroups of patients differing in the expression of genes associated with cytokine signaling, immune regulation and extracellular matrix reorganization. One of the two subgroups identified (subgroup A) is associated with a high expression of protumorigenic, immunosuppressive and clinically unfavorable markers, including IL-6, IL-10, CD163 and PCOLCE2. By contrast, the second subgroup is characterized by the upregulation of genes linked to IFN signaling and associated with a longer survival. Expression of this IFN-related signature also showed a striking link to a longer survival, and IFNγ abrogated the inhibitory effect of ovarian cancer ascites on the inducibility of IL-12 in cultured macrophages. As IL-12 is a key mediator of a cytotoxic immune response, this finding provides a possible explanation for the link of the IFN signaling-associated signature B to ovarian cancer survival.

## Additional files

Additional file 1: Supplemental Table.
**Table S1: **Patients data (RNA-Seq). (XLSX 49 kb)
Additional file 2: Supplemental Datasets S1-S10.
**Dataset S1:** Complete RNA-Seq data. **Dataset S2:** EdgeR results. **Dataset S3:** Signature A expression data. **Dataset S4: **Signature B expression data. **Dataset S5:** Cluster I expression data. **Dataset S6:** Cluster II expression data. **Dataset S7:** Cluster III expression data. **Dataset S8:** Common cluster I / signature A genes. **Dataset S9: **Common cluster III / signature B genes. **Dataset S10:** PRECOG z-scores for signature A and B genes. (XLSX 6898 kb)
Additional file 3: Supplemental Figures S1-S8.
**Figure S1: **Inverse association of PCOLCE2 expression with high-grade serous ovarian cancer survival (RFS). **Figure S2:** Hierarchial clustering of coexpressed high variance genes. **Figure S3: **Venn diagram showing the overlaps of the the upstream regulators gene sets identified in Fig. 3C. **Figure S4: **Subgroup-selective expression of signature genes. **Figure S5:** Association of signature A (top) and signature B (bottom) with ovarian cancer survival (OS). **Figure S6:** Association of the ECM remodeling-linked genes of signature A with high-grade serous ovarian cancer survival. **Figure S7: **Association of tumor-infiltrating host cells with high-grade serous ovarian cancer survival (OS). **Figure S8: **Expression of type I IFN genes in different cell types present in ovarian cancer ascites. (PDF 1015 kb)

## Abbreviations

GO: Gene ontology
IFN: Interferon
IL: Interleukin
LPS: Lipopolysaccharide
MDM: Monocyte-derived macrophage
OS: Overall survival
PCA: Principal component analysis
PCOLCE2: Procollagen C-endopeptidase enhancer 2
RFS: Relapse-free survival
RNA-Seq: RNA sequencing
TAM: Tumor-associated macrophage
TNF: Tumor necrosis factor α
TPM: Transcripts per million
