# Supplementary material for: Interferon signaling in ascites-associated macrophages is linked to a favorable clinical outcome in a subgroup of ovarian carcinoma patients
Source: BMC Genomics. 2017 Mar 21;18:243. doi: 10.1186/s12864-017-3630-9 (PMC5359932; doi:10.1186/s12864-017-3630-9)
Supplement: Additional file 3: Supplemental Figures S1-S8. — Figure S1: Inverse association of PCOLCE2 expression with high-grade serous ovarian cancer survival (RFS). Figure S2: Hierarchial clustering of coexpressed high variance genes. Figure S3: Venn diagram showing the overlaps of the the upstream regulators gene sets identified in Fig. 3C. Figure S4: Subgroup-selective expression of signature genes. Figure S5: Association of signature A (top) and signature B (bottom) with ovarian cancer survival (OS). Figure S6: Association of the ECM remodeling-linked genes of signature A with high-grade serous ovarian cancer survival. Figure S7: Association of tumor-infiltrating host cells with high-grade serous ovarian cancer survival (OS). Figure S8: Expression of type I IFN genes in different cell types present in ovarian cancer ascites. (PDF 1015 kb) [file 12864_2017_3630_MOESM3_ESM.pdf]

Additional file 3

Interferon signaling in ascites-associated macrophages is associated with a favorable clinical outcome in a subgroup of ovarian carcinoma patients

Till Adhikary[1], Annika Wortmann[1], Florian Finkernagel[1], Sonja Lieber[1], Andrea Nist[2], Thorsten Stiewe[2], Uwe Wagner[3], Sabine Müller-Brüsselbach[1], Silke Reinartz[3] and Rolf Müller[1]*

[1]Institute of Molecular Biology and Tumor Research (IMT), Center for Tumor Biology and Immunology (ZTI), Philipps University, Marburg, Germany

[2]Genomics Core Facility, Center for Tumor Biology and Immunology (ZTI), Philipps University, Marburg, Germany

[3]Clinic for Gynecology, Gynecological Oncology and Gynecological Endocrinology, Center for Tumor Biology and Immunology (ZTI), Philipps University, Marburg, Germany

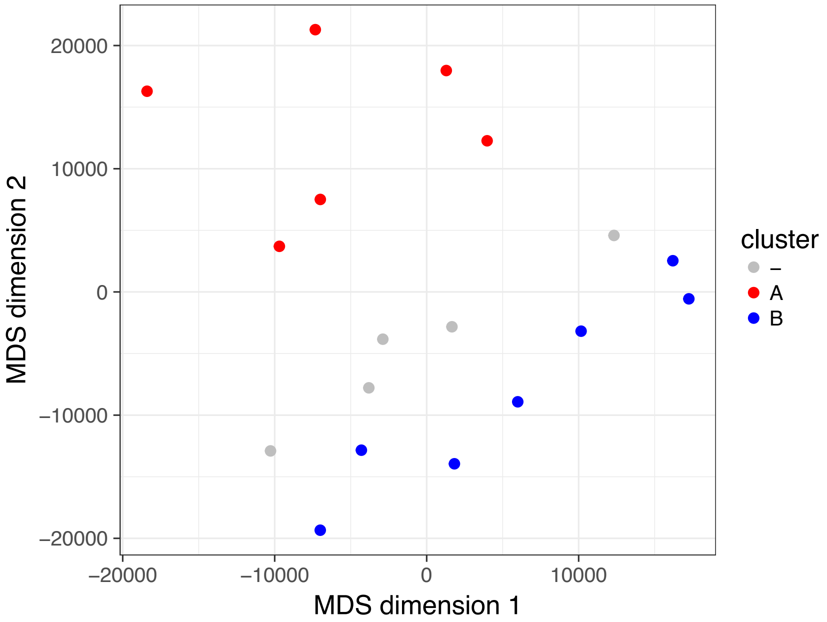

Figure S1.

Multidimensional scaling analysis of TAM samples.

Clusters A and B refer to the clusters identified by PCA in Fig. 2A. Analysis was performed as for PCA except that the *sklearn.manifold.MDS* function *mds.fit_transform ()* was used.

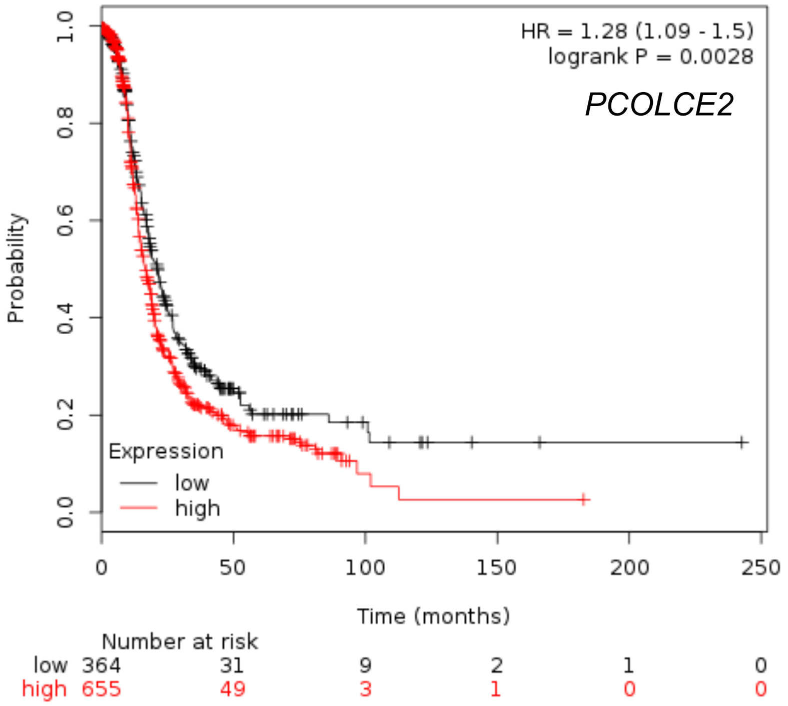

Figure S2.

Inverse association of *PCOLCE2* expression with high-grade serous ovarian cancer survival (RFS).

The Kaplan-Meier plot was generated by the KM plotter online tool (http://kmplot.com).

Reference

Gyorffy B, Lanczky A, Szallasi Z. Implementing an online tool for genome-wide validation of survival-associated biomarkers in ovarian-cancer using microarray data from 1287 patients. *Endocr Relat Cancer* 2012;**19**: 197-208.

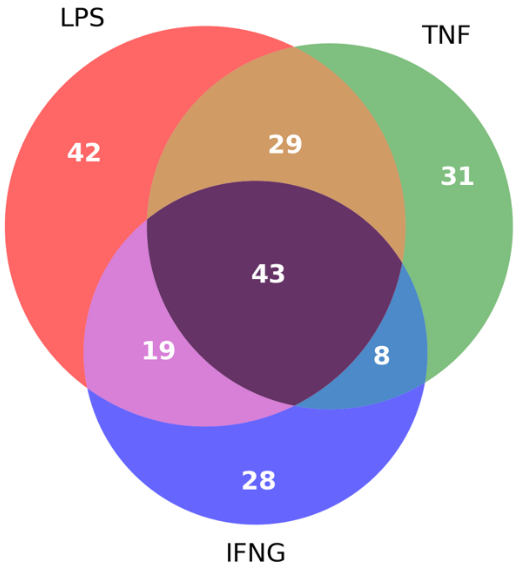

Figure S3.

Venn diagram showing the overlaps of the the upstream regulators gene sets identified in Fig. 3C.

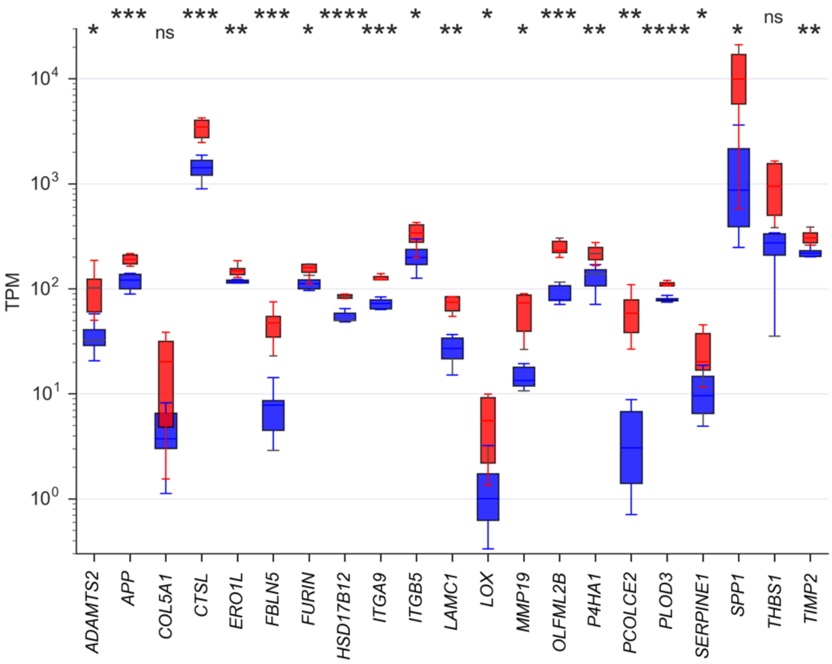

Figure S4.

Subgroup-selective expression of signature genes.

Expression of the ECM-associated genes of signature A identified by GO enrichment analysis (Fig. 3C) in subgroup A and B TAMs. Boxes show the upper and lower quartiles, whiskers the 95% CI, and horizontal lines the median.

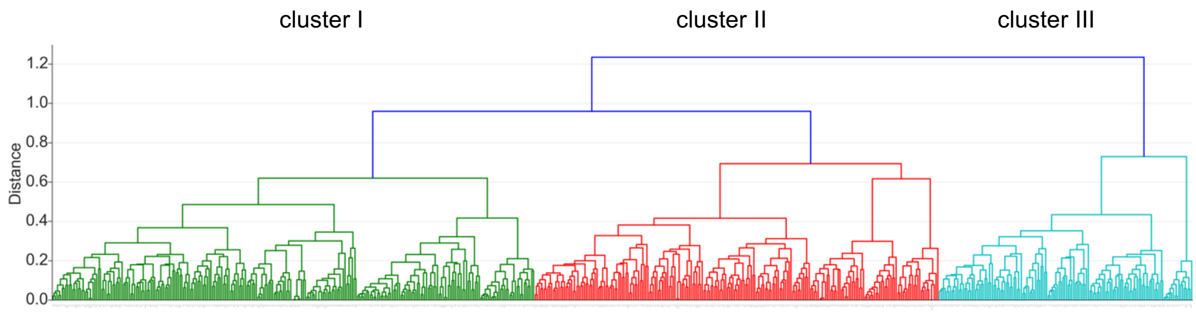

Figure S5.

Hierarchial clustering of coexpressed high variance genes.

Hierarchial clustering was performed with all TAM samples (n=18) using the *scipy.cluster.hierarchy* functions *linkage (method="weighted", metric="correlation")* and *dendrogram (truncate_mode = "none", color_threshold=1)*.

Green: cluster I; red: cluster II; cyan: cluster III.

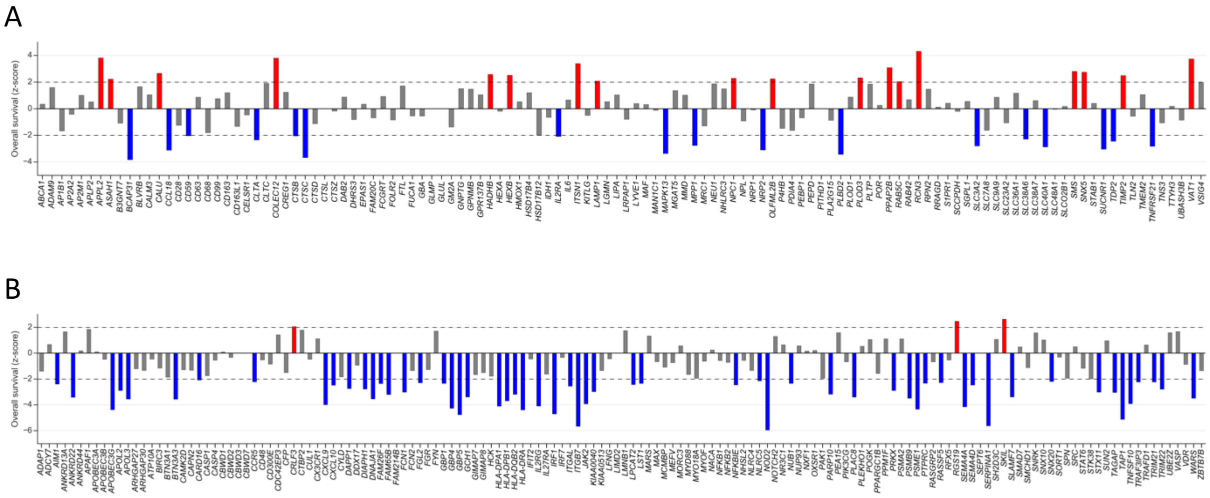

Figure S6.

Association of signature A (top) and signature B (bottom) with ovarian cancer survival (OS).

Data (z-scores) were obtained from the PRECOG database, which contains the results of a meta-analysis of 1763 patients from 12 studies.

Reference

Gentles AJ, Newman AM, Liu CL, Bratman SV, Feng W, Kim D, Nair VS, Xu Y, Khuong A, Hoang CD, Diehn M, West RB, et al. The prognostic landscape of genes and infiltrating immune cells across human cancers. *Nature medicine* 2015;**21**: 938-45.

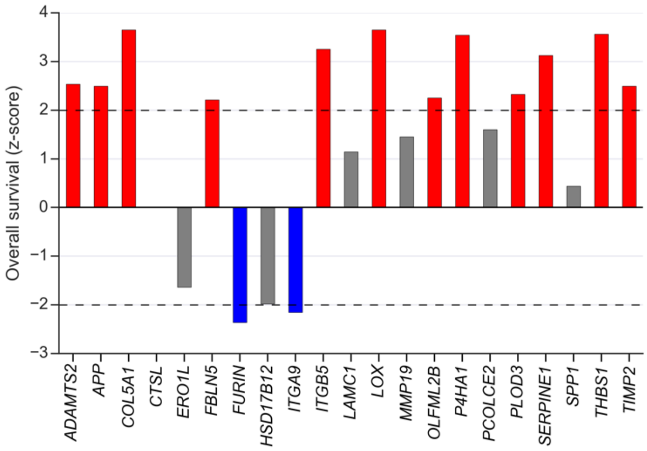

Figure S7.

Association of the ECM remodeling-linked genes of signature A with high-grade serous ovarian cancer survival.

PRECOG data, details in Figure S5.

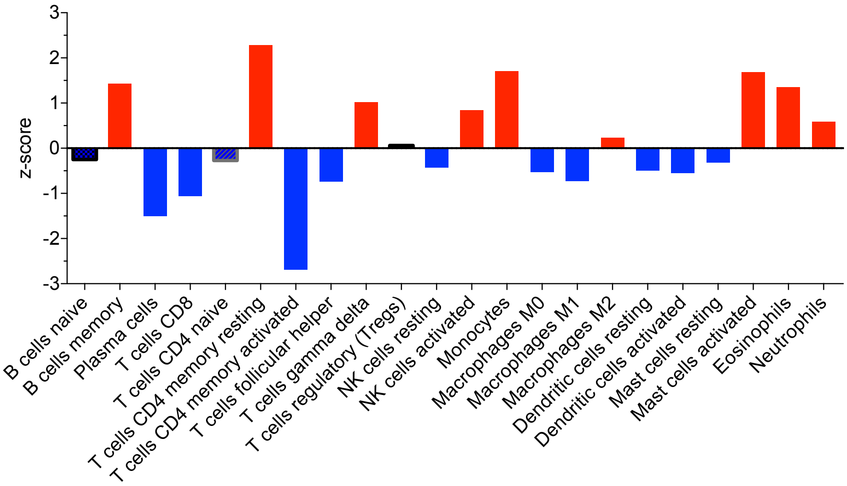

Figure S8.

Association of tumor-infiltrating host cells with high-grade serous ovarian cancer survival (OS).

Host cell infiltration inferred from RNA expression data using CIBERSORT was associated with OS. The data show no significant association of OS with myeloid cells, but a trend towards a shorter OS for monocytes.

Reference

Newman AM, Liu CL, Green MR, Gentles AJ, Feng W, Xu Y, Hoang CD, Diehn M, Alizadeh AA. Robust enumeration of cell subsets from tissue expression profiles. Nat Methods 2015;12: 453-7.

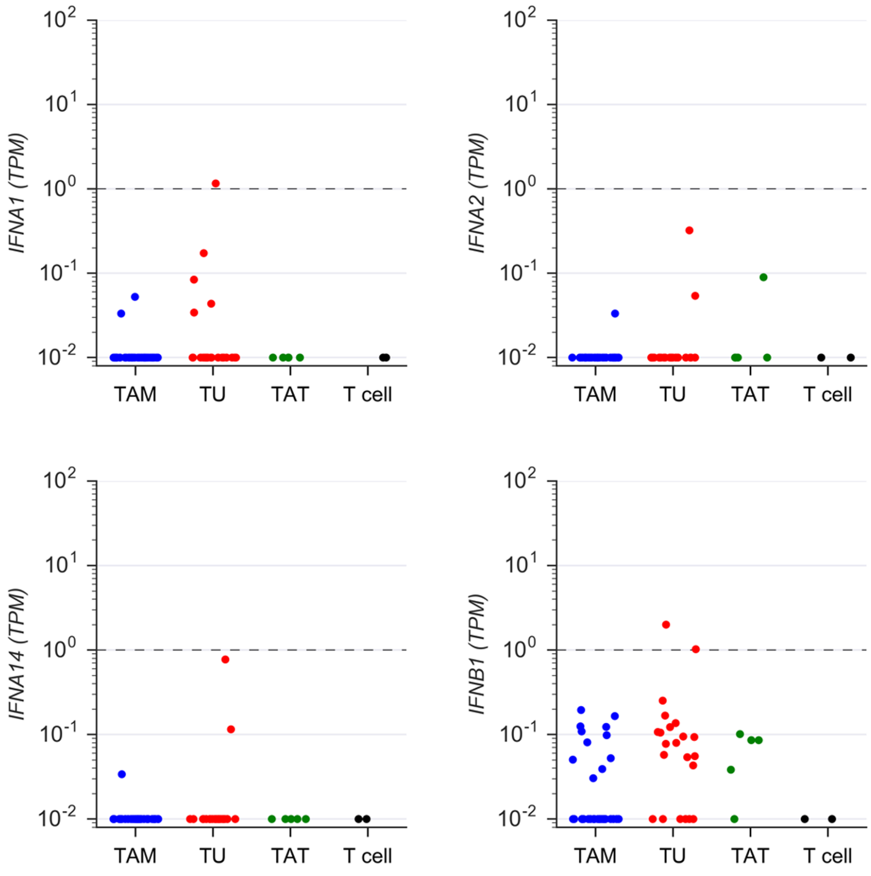

Figure S9.

Expression of type I IFN genes in different cell types present in ovarian cancer ascites.

Expression of type I IFN genes showing a significant association with OS (Fig. 6A; blue bars) in TAM (n=33), tumor cell (n=22) and TAT (n=5) samples from ovarian carcinoma ascites, and in $CD3^+$ T cells from healthy donors (n=2). Each dot represents an individual sample (see Dataset S1 for details).
